# Performance of an Influenza Rapid Test in Children in a Primary Healthcare Setting in Nicaragua

**DOI:** 10.1371/journal.pone.0007907

**Published:** 2009-11-19

**Authors:** Aubree Gordon, Elsa Videa, Saira Saborio, Roger López, Guillermina Kuan, Arthur Reingold, Angel Balmaseda, Eva Harris

**Affiliations:** 1 Division of Epidemiology, School of Public Health, University of California, Berkeley, California, United States of America; 2 Fogarty International Center, National Institutes of Health, Bethesda, Maryland, United States of America; 3 Sustainable Sciences Institute, Managua, Nicaragua; 4 Departamento de Virología, Centro Nacional de Diagnóstico y Referencia, Ministry of Health, Managua, Nicaragua; 5 Centro de Salud Sócrates Flores Vivas, Ministry of Health, Managua, Nicaragua; 6 Division of Infectious Diseases and Vaccinology, School of Public Health, University of California, Berkeley, California, United States of America; Washington University School of Medicine, United States of America

## Abstract

**Background:**

Influenza is major public health threat worldwide, yet the diagnostic accuracy of rapid tests in developing country settings is not well described.

**Methodology/Principal Findings:**

To investigate the diagnostic accuracy of the QuickVue Influenza A+B test in a primary care setting in a developing country, we performed a prospective study of diagnostic accuracy of the QuickVue Influenza A+B test in comparison to reverse transcriptase-polymerase chain reaction (RT-PCR) in a primary healthcare setting in children aged 2 to 12 years in Managua, Nicaragua. The sensitivity and specificity of the QuickVue test compared to RT-PCR were 68.5% (95% CI 63.4, 73.3) and 98.1% (95% CI 96.9, 98.9), respectively, for children with a fever or history of a fever and cough and/or sore throat. Test performance was found to be lower on the first day that symptoms developed in comparison to test performance on days two or three of illness.

**Conclusions/Significance:**

Our study found that the QuickVue Influenza A+B test performed as well in a developing country primary healthcare facility setting as in developed country settings.

## Introduction

Influenza is a major health threat throughout the world [1], yet many countries lack even rudimentary influenza surveillance systems [2], [3]. Furthermore, influenza surveillance in developing countries often includes only major cities due to limited laboratory resources in outlying areas and poor infrastructure, such as lack of electrical power. Consequently, data are lacking about the types of influenza viruses that are circulating and the burden of influenza-related disease in the developing world.

Logistical constraints in developing countries largely dictate the types of laboratory tests available for use in influenza surveillance. While reverse transcriptase-polymerase chain reaction (RT-PCR) and viral isolation are the preferred diagnostic methods, rapid influenza tests are valuable for their ease of use and laboratory-independence. In the developed world, rapid influenza tests have been evaluated in both laboratory and clinical settings, yielding sensitivities of 45–90% and specificities of 86–100% [4], [5], [6], [7], [8], [9], [10] in comparison to viral isolation and RT-PCR. In developing countries, the performance characteristics of rapid influenza tests are not well documented in primary health care settings, where infrastructure is unreliable, conditions are often hot and humid, and staff may have little or no experience with the collection of respiratory samples, yet where rapid tests may aid in influenza surveillance.

Historically, viral isolation has been regarded as the “gold standard” for diagnosis of influenza infection. However, recently a number of studies have demonstrated the superior sensitivity of RT-PCR for the detection of influenza viruses [11], [12], [13], [14], and influenza experts have recommended that RT-PCR be regarded as the new diagnostic standard [14], [15]. Surveillance systems in developed countries and some developing countries have switched to using RT-PCR as the primary laboratory diagnostic test, with viral isolation performed primarily on RT-PCR-positive specimens. For this reason, the QuickVue rapid test was compared to RT-PCR in this study.

To examine the diagnostic accuracy of the QuickVue Influenza A+B test in comparison to RT-PCR in children treated in a primary healthcare facility in a developing country, we performed a prospective study in Managua, Nicaragua and report the results here.

## Methods

### Ethics Statement

This study was approved by the institutional review boards at the University of California (UC) Berkeley and the Nicaraguan Ministry of Health. Written informed consent was obtained from the parents or guardians of all participants.

### Study Population and Site

This study was performed as part of the Nicaraguan Influenza Cohort Study (NICS), a prospective community-based cohort study of approximately 3,800 children 2–14 years old in Managua, Nicaragua [16]. Healthy children aged 2–12 years residing in the study area were enrolled beginning in June 2007. Children were eligible to remain in the study through the age of 14 or until they moved from the study area. To maintain the age structure of the cohort, new two-year-olds were recruited in July-September of each year. The primary study site was the Health Center Socrates Flores Vivas (HCSFV), a public primary health care facility in the capital of Nicaragua. Respiratory specimens were collected and the rapid test was performed in the HCSFV's clinical laboratory. A number of measures were taken to encourage participants to attend the HCSFV at the first sign of illness. These measures included: 1) at enrollment, parents agreed to bring their child to the health center at the first sign of illness; 2) participants were provided with free healthcare 24 hours a day, 365 days a year; 3) participants were provided with rapid diagnostic testing free of charge; and 4) a study ambulance was available at all times at the heath center. Children who required hospitalization or tertiary-level care were transferred to a hospital by study staff. In yearly participation surveys, 1.7% to 2.5% of participants reported attending a healthcare provider outside of the study.

### Procedures

All specimens collected between January 1, 2008 and December 31, 2008 from children in the NICS who presented with a fever, or history of fever or feverishness, and cough and/or sore throat within five days of symptom onset were included in the study. Information from all medical appointments was systematically collected using standardized data collection forms. Data included age, sex, two measurements of temperature, date of fever onset, and other symptoms. Upon presentation with testing criteria, children were randomly selected for respiratory testing using a random number generator and then referred to the HCSFV clinical laboratory. There, a nasal swab was first collected for the rapid test, QuickVue Influenza A+B, using the swab provided in the kit. Then nasal and throat Dacron swabs were collected and placed into one tube containing three mL of viral transport medium for RT-PCR and viral culture. The QuickVue test was performed onsite immediately. Samples for RT-PCR and viral isolation collected on workdays (Monday–Saturday) were kept at 4°C and processed within 16 hours. Samples collected on Sundays and holidays were stored for up to 48 hours prior to processing.

QuickVue Influenza A+B is a lateral-flow immunoassay that detects and differentiates between influenza A and B. Tests were performed according to the manufacturer's instructions. Tests were read after exactly ten minutes and were considered negative if, at ten minutes, a pink line had not appeared to indicate a positive influenza A or B result. Tests were considered invalid if the blue control line did not appear or if the background color made it impossible to read the test result. Invalid tests were repeated once.

### Laboratory Methods

Samples were transported to the National Virology Laboratory at the Nicaraguan Ministry of Health, where RNA was extracted from nasal swabs using the QIAamp® Viral RNA Mini Kit. (Qiagen Sciences, Germantown, MD) Influenza viruses A and B were amplified using Promega Access RT-PCR (Promega Corporation, Madison, WI) and typed in-house according to a protocol provided by the Centers for Disease Control and Prevention (CDC). Laboratory technicians were trained in how to perform RT-PCR, including attendance at influenza testing workshops held by the CDC and World Health Organization and hands-on training by study personnel from UC Berkeley. Laboratory technicians performing RT-PCR testing were blinded to the results of the rapid test and to the patients' clinical presentation.

### Rapid Test Training

To replicate the typical training resources that would be available if rapid tests were widely distributed in a developing country setting, a 1.5-hour training session was held. This training consisted of a short presentation explaining the symptoms and burden of influenza, influenza surveillance, collection of nasal swabs, principles of the QuickVue rapid test, and instructions on how to perform the test. Laboratory technicians then practiced taking one nasal swab specimen and performing a rapid test. Prior to the training session, technicians had never performed an influenza rapid test and many had little or no experience collecting nasal swab specimens.

### Statistical Analyses

The main outcomes of this study were the sensitivity, specificity, positive predictive value (PPV), and negative predictive value (NPV) of the QuickVue Influenza A+B test compared with RT-PCR. Agreement between tests was calculated using the kappa statistic. Sub-analyses were performed to examine test performance stratified by testing criteria, day of presentation, age, and sex. All statistical analyses were performed using STATA 10.1 (StataCorp, College Station, Texas).

## Results

**Figure 1:**
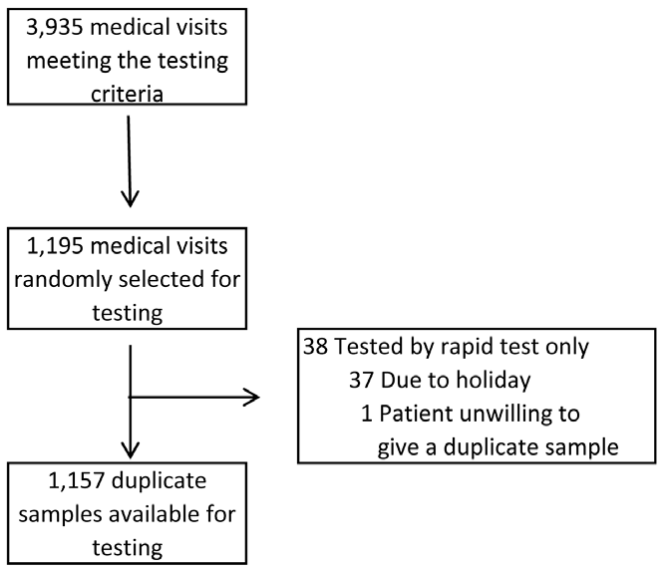
Flowchart of sample selection, Managua, Nicaragua, 2008. Flowchart diagramming the selection of samples used to analyze the performance of the QuickVue Influenza A+B rapid test. Due to complications related to transport and sample separation, samples (n = 37) collected during holiday periods lasting longer than 3 days did not undergo RT-PCR and therefore were not included in this study.

Between January 1, 2008, and December 31, 2008, 3,916 children participated in the cohort study. During this period, study participants attended 13,666 medical visits, of which 3,935 (29%) met the testing criteria. Of these, 1,195 (30%) were randomly selected for influenza testing, and 1,157 duplicate specimens (97%) were collected from 989 children and tested by both rapid test and RT-PCR, while 38 children received a rapid test only (Figure 1). Demographic data for the children that contributed samples are presented in Table 1. In total, 359 samples (31%) tested positive for influenza by RT-PCR, with 224 (19%) testing positive for influenza A and 135 (12%) for influenza B. Of the 74 influenza A-positive samples that underwent subtyping by RT-PCR, 73 were positive for seasonal influenza A H1N1 and one for influenza A H3N2. The QuickVue test detected virus in 246 influenza RT-PCR-positive samples, including 146 influenza A-positive samples and 100 influenza B-positive samples. One invalid QuickVue test result was repeated and found to be negative for influenza.

**Table 1:** Demographic description of study participants, Managua, Nicaragua, 2008.

	All cases n = 1,157	Cases with influenza-like illness n = 578
Sex		
Female	581 (50.2)	301 (52.1)
Age (years)		
2	84 (7.3)	42 (7.3)
3	169 (14.6)	92 (15.9)
4	166 (14.4)	79 (13.7)
5	158 (13.7)	76 (13.2)
6	129 (11.2)	75 (13.0)
7	113 (9.8)	63 (10.9)
8	113 (9.8)	52 (9.0)
9	79 (6.8)	34 (5.9)
10	53 (4.6)	23 (4.0)
11	53 (4.6)	26 (4.5)
12	36 (3.1)	15 (2.6)
13	4 (0.4)	1 (0.2)

Data on the agreement of RT-PCR and rapid test are presented in Table 2. Overall, the sensitivity and specificity of the QuickVue test compared to RT-PCR for influenza A or B were 68.5% (95% CI 63.4, 73.3) and 98.1% (95% CI 96.9, 98.9), respectively. The corresponding PPV was 93.9% (95% CI 90.3, 96.5), and the NPV was 88.1% (95% CI 85.9, 90.1) (Table 3). The sensitivity and specificity of the test in children under six and in those aged six and over were similar (data not shown).

**Table 2:** Agreement between the QuickVue Influenza A+B test and RT-PCR, Managua, Nicaragua, 2008.

Patients with fever or a history of fever and cough and/or sore throat (n = 1,157)					
		RT-PCR		Agreement (%)	Kappa (95% CI)
		+	−		
QuickVue	+	246	15	88.9	0.72 (0.67, 0.76)
	−	113	783		
Patients with a temperature ≥37.8°C and cough and/or sore throat (n = 578)					
		RT-PCR		Agreement (%)	Kappa (95% CI)
		+	−		
QuickVue	+	189	6	86.3	0.72 (0.65, 0.77)
	−	73	310		

**Table 3:** Performance of the QuickVue Influenza A+B Test, Managua, Nicaragua, 2008.

	Number Positive	Sensitivity		Specificity		Positive Predictive Value		Negative Predictive Value	
		(%)	(95% CI)	(%)	(95% CI)	(%)	(95% CI)	(%)	(95% CI)
Children presenting with a fever or reported fever and cough and/or sore throat (n = 1,157)									
Influenza	359	68.5	(63.4, 73.3)	98.1	(96.9, 98.9)	94.3	(90.7, 96.7)	87.4	(85.0, 89.5)
Influenza A	224	65.2	(58.5, 71.4)	99.1	(98.3, 99.6)	94.8	(90.0, 97.7)	92.2	(90.4, 93.8)
Influenza B	135	74.1	(65.8, 81.2)	99.1	(98.3, 99.6)	91.7	(84.9, 96.2)	96.7	(95.4, 97.7)
Children presenting with a fever ≥37.8°C and cough and/or sore throat (n = 578)									
Influenza	262	72.1	(66.3, 77.5)	98.1	(95.9, 99.3)	96.9	(93.4, 98.9)	80.9	(76.6, 84.8)
Influenza A	169	69.8	(62.3, 76.6)	99.3	(97.9, 99.8)	97.5	(92.9, 99.5)	88.8	(85.6, 91.6)
Influenza B	93	76.3	(66.4, 84.5)	99.2	(97.9, 99.8)	94.7	(86.9, 98.5)	95.6	(93.5, 97.2)

Limiting the analysis to the 578 samples from patients who met the CDC definition of influenza-like illness, defined as a fever of ≥37.8°C plus cough or sore throat, resulted in a slightly higher sensitivity for the rapid test but did not significantly change the overall test performance (Tables 2 and 3). In this sub-analysis, the sensitivity of the rapid test was 72.1% (95% CI 66.3, 77.5) and the specificity was 98.1% (95% CI 95.9, 99.3). Performance of the rapid test by day of presentation was examined both for the entire study population and for the subgroup meeting the definition of ILI. In both analyses, the rapid test had a lower sensitivity on the day of symptom onset and on the fourth day of illness when compared to the sensitivity on day two or three of illness (Table 4).

**Table 4:** Performance of the QuickVue Influenza A+B Test in children by day post-onset of symptoms, Managua, Nicaragua, 2008.

	Sample Number	Number Positive	Sensitivity		Specificity		Positive Predictive Value		Negative Predictive Value	
			(%)	(95% CI)	(%)	(95% CI)	(%)	(95% CI)	(%)	(95% CI)
Patients with fever or a history of fever and cough and/or sore throat (n = 1,157)										
Day 1	263	79	51.9	(40.4, 63.3)	98.4	(95.3, 99.7)	93.2	(81.3, 98.6)	82.6	(77.0, 87.4)
Day 2	578	189	75.1	(68.3, 81.1)	97.9	(96.0, 99.1)	94.7	(89.8, 97.7)	89	(85.7, 91.8)
Day 3	211	66	74.2	(62.0, 84.2)	97.9	(94.1, 99.6)	94.2	(84.1, 98.8)	89.3	(83.4, 93.6)
Day 4	90	19	57.9	(33.5, 79.7)	98.6	(92.4, 100)	91.7	(61.5, 99.8)	89.7	(80.8, 95.5)
Patients with a fever ≥37.8°C and cough and/or sore throat (n = 578)										
Day 1	182	62	59.7	(46.4, 71.9)	97.5	(92.9, 99.5)	92.5	(79.6, 98.4)	82.4	(75.1, 88.3)
Day 2	284	147	76.9	(69.2, 83.4)	98.5	(94.8, 99.8)	98.3	(93.9, 99.8)	79.9	(73.0, 85.6)
Day 3	78	39	79.5	(63.5, 90.7)	97.4	(86.5, 99.9)	96.9	(83.8, 99.9)	82.6	(68.6, 92.2)
Day 4	26	11	54.5	(23.4, 83.3)	100	(78.2, 100)	100	(54.1, 100)	75.0	(50.9, 91.3)

Note: Day 1 refers to the day of symptom onset.

## Discussion

Our study found that the QuickVue Influenza A+B test performed with a moderate sensitivity (68.5%) and high specificity (98.1%) in comparison to RT-PCR in a developing country primary care setting. Laboratory technicians found the test easy to use and needed minimal training to adequately perform the test.

In the US and other developed countries, the QuickVue Influenza A+B rapid test has been found to have a sensitivity of 67%–85% and specificity of 97%–100% [8], [17]. However, one recent study conducted in three sites in the US found the test performed with a significantly lower sensitivity (19%–32%)[18]. One study performed in a developing country reported the QuickVue test to have a sensitivity and specificity of 71% and 98%, respectively, in patients aged one month to 85 years in hospital outpatient clinics in Thailand [15]. As most of these prior studies included only patients meeting the CDC/WHO definition of ILI, it is more appropriate to compare the results of our sub-analysis limited to children with ILI to these studies. Thus, the sensitivity of 72.1% (95% CI 66.3, 77.5) and specificity of 98.1% (95% CI 95.9, 99.3) we observed are well within the ranges of the sensitivities and specificities previously reported.

Importantly, a substantial number of participants (23%) presented on the first day of illness, and the sensitivity of the rapid test was found to be lower among children who presented and were tested on the first day. Few studies of rapid tests have captured data on these early cases. In a study in Hong Kong, researchers examined the performance of the QuickVue rapid test and did not find a significant difference in the first 24 hours following symptom onset [19]. One possible explanation for our differing results is that the Hong Kong study population was very different from ours. Subjects were predominantly adults and were recruited because they were seeking medical care at an outpatient clinic, and therefore might have been sicker and hence have a higher viral load than our study subjects at the time of testing. In a recent meta-analysis of influenza volunteer challenge studies, symptoms were reported in the first 24 hours following virus inoculation, a day before peak viral load and two days before the peak of symptoms [20]. It is possible that early in illness, children are symptomatic at relatively low viral loads, leading to the lower sensitivity we report on day 1. More studies are needed to elucidate the natural history of influenza infection in children.

The large number of children presenting so early resulted from the cohort study protocol, in which families of participants were asked to bring in their children at the first sign of illness. However, persons with influenza typically do not present until several days into the illness. Therefore, it is likely that the sensitivity and resulting positive predictive values we observed are underestimates of the test's performance in the clinical setting, since it is less common for patients to present on the day of symptom onset. However, during an influenza pandemic, patients may present very early following symptom onset; thus, it is imperative to evaluate the performance of any rapid test in detecting influenza very early after onset of illness.

One of the limitations of this study is the age range of the patients. Because the study was restricted to children aged 2–14 years, the sensitivities and specificities we found apply only to this group. However, because the rapid test performed at approximately the same level in this study as in studies performed in children in developed country settings, the rapid test may well perform at the same level across all age ranges in developing country settings as observed in studies conducted in developed countries. Another limitation of this study is the use of RT-PCR as the gold standard. However, considering the very high (∼98%) specificity of the rapid test in this study and the fact that RT-PCR is substantially more sensitive than viral isolation, it is unlikely that use of RT-PCR alone, instead of a combination of RT-PCR and viral isolation, significantly affected the results.

In conclusion, the rapid influenza test we used performed well in a primary healthcare setting in Managua, Nicaragua. Clinical laboratory staff needed minimal training to correctly perform the test and found the test easy to perform. Further evaluation of rapid tests in developing countries is needed to determine the feasibility of using these tests to augment existing surveillance systems and for quick deployment in the event of an influenza pandemic, as well as for use in clinical decision-making.
